# Quantifying the effects of aging and urbanization on major gastrointestinal diseases to guide preventative strategies

**DOI:** 10.1186/s12876-018-0872-1

**Published:** 2018-10-03

**Authors:** Liu Hui

**Affiliations:** Department of Clinical Immunology, Dalian Medical University, Dalian, 116044 People’s Republic of China

**Keywords:** Gastrointestinal disease, Cancer, Aging, Urbanization, Categorization, Epidemiology

## Abstract

**Background:**

This study aimed to quantify the effects of aging and urbanization on major gastrointestinal disease (liver cirrhosis, hepatitis B, diarrhea, liver cancer, stomach cancer, pancreas cancer, hepatitis C, esophagus cancer, colon/rectum cancer, gastrointestinal ulcers, diabetes, and appendicitis).

**Methods:**

We accessed 2004 and 2011 mortality statistics from the most developed cities and least developed rural areas in China using a retrospective design. The relative risk of death associated with urbanization and age was quantified using Generalized linear model (the exp.(B) from model is interpreted as the risk ratio; the greater the B, the greater the impact of urbanized factors or aging factor or effect of aging factor with urbanization). The interaction between region (cities and rural areas) and age was considered as indicator to assess role of age in mortality with urbanization.

**Results:**

Greater risk of disease with urbanization were, in ascending order, for diabetes, colon/rectum cancer, hepatitis C and pancreas cancer. Stronger the effect of aging with urbanization were, in ascending order, for stomach cancer, ulcer, liver cancer, colon/rectum cancer, pancreas cancer, diabetes, hepatitis C, appendicitis and diarrhea. When the effects of aging and urbanization on diseases were taken together as the dividing value, we were able to further divide the 12 gastrointestinal diseases into three groups to guide the development of medical strategies.

**Conclusions:**

It was suggested that mortality rate for most gastrointestinal diseases was sensitive to urbanization and control of external risk factors could lead to the conversion of most gastrointestinal disease.

## Background

Gastrointestinal disease refers to disease involving the gastrointestinal tract, including disease of the esophagus, stomach, small intestine, large intestine and rectum, and the accessory organs of digestion, liver, gallbladder and pancreas. Gastrointestinal disease may have many pathomechanisms can be infectious or chronic, and involve acute or chronic inflammation, or cancer.

Global urbanization, which has continued to increase since the arrival of the industrial revolution, results in alteration to and loss of natural habitats. A sedentary lifestyle, higher-kilojoule food intake, a decrease in exposure to sunlight, and increasingly stressful conditions have all been associated with increasing urbanization, which, in turn, may be impacting on prevalence of gastrointestinal disease. Urbanization leads to many challenges, not only for global health, but also specifically to the field of gastrointestinal disease [1–3]. To identify and implement medical strategies, it is necessary to explore how rapid urbanization may be affecting the burden of gastrointestinal disease in growing economies. To date, there have been no comprehensive studies into the quantitative effects of urbanization on gastrointestinal disease.

China has a relatively simple ethnic composition and a large land area, and is presently undergoing rapid urbanization [4–6]. Economic growth has had a profound impact on the health of its citizens and levels of medical care; however, because of an unbalanced economy, there is considerable disparity between regions. China, therefore, is an ideal model for assessing patterns of disease occurrence, development and changes with this modernization process [7–9].

Life expectancy will increase with urbanization [10–12]. Greater human longevity and an increasingly elderly population might also be important reasons for changes in the spectrum of gastrointestinal disease. Therefore, this study focused on the impact of urbanization and the aging process on gastrointestinal disease. We found that these two properties varied significantly in different gastrointestinal diseases (diarrhea, viral hepatitis B, viral hepatitis C, esophagus cancer, stomach cancer, colon/rectum cancer, liver cancer, pancreas cancer, diabetes, gastrointestinal ulcers, liver cirrhosis and appendicitis). To inform medical strategies, we also categorized the 12 major gastrointestinal diseases listed above, based on the quantitative effects of age and urbanization.

## Methods

### Original data

We obtained raw data from the 2004 and 2011 data sets of the National Disease Mortality Surveillance System [13, 14]. These data were obtained from people living in the most undeveloped rural areas (rural) in 2004 and the most developed cities (urban) in 2011 for keeping considerable disparity between regions. The definition of urbanization and rural areas was according to administrative regions of China; name with city or district in city (a large majority of the population is not engaged in agriculture and/or fishing) was defined as urban and others were rural areas (Table 1) [13, 14]. The causes of death must be recorded in patient’s place of household registration. The mortality due to nutritional deficiencies was 1.32/0.1 million individuals (standard rate) in the rural population and 0.80/0.1 million individuals (standard rate) in the urban population and; the life expectancy at birth was 73.90 years in the rural areas and 79.53 years in the urban areas, respectively [13, 14]. The underlying causes of death (the disease which initiated the train of morbid events leading directly to death) were classified according to International Classification of Diseases (ICD)-10 codes [15] to determine the mortality statistics. Rate of objective diagnosis (as evidenced by laboratory, pathologic, imaging and/or surgical intervention findings) was 90.76% in rural areas and that was 92.00% in urban areas [13, 14]. The raw data are presented in Table 2 and Table 3.

**Table 1:** The name of urban and rural area/regions where data collected in China

Cities	Cities	Rural areas	Rural areas
Dongcheng in Beijing	Tongxiang	Balimyouqi	Xiangyunxian
Tongzou in Beijing	Wucheng Jinhua	Kailuxian	Lanpingxian
Hongqiao in Tianjin	Meijie in Sanming	Suniteyouqi	Milinxian
Kaiping in Tangshan	Jianou	Binyangxiang	Naidongxian
Haigang in Qinhuangdao	Jiaocheng in Ningde	Hepuxian	Jiangzixian
Wuan	Shibei in Qingdao	Lingyunxian	Meixian
Qiaodong in Zhangjiakou	Xuecheng in Zaozhang	Luochengxian	Luochuanxia
Xinceng in Shenyang	Zifu in Yantai	Dazuxian	Hanyinxiangn
Shahekou in Dalian	Penglai	Zizhongxian	Jingtaixian
Qianshan in Anshan	Gaomi	Xichongxian	Lintanxian
Fengcheng	Laicheng in Laiwu	Shuangyuanxian	Pinganxian
Luwan in Shanghai	Lichang inQingdao	Kangdingxian	Menyuanxian
Songjiang in Shanghai	Xiuyue in Guangzhou	Yuexixian	Xinhexian
Pukou in Nanjing	Nanxiong	Meitanxian	Shachexian
Yunlong in Xuzou	Sihui	Yupingxian	Hetianxian
Wuzhong in Suzhou	Shanwei	Dushanxian	Xinyuanxian
Zhangjiagang	Yunfu	Tonghaixian	_
Xiacheng in Hangzou	Meilian in haikou	Guangnanxian	_
Fenghua	_	Menglaxian	_

**Table 2:** Age-stratified number of deaths from major gastrointestinal diseases in 2004 in underdeveloped rural areas of China [13]

Age	Gastrointestinal diseases												All cause	Survival
	A	B	C	D	E	F	G	H	I	J	K	L		
0-	180	0	0	0	0	0	1	0	0	0	2	0	3038	173,744
1-	88	6	0	0	0	0	2	0	2	0	1	1	1186	792,643
5-	11	4	0	0	0	1	3	0	2	0	3	1	597	1,145,068
10-	3	5	0	0	0	1	6	0	2	1	2	0	557	1,437,804
15-	6	2	0	0	1	4	8	1	1	1	3	2	948	1,247,497
20-	3	5	0	4	5	8	15	0	1	5	8	3	1213	1,161,369
25-	2	13	0	2	17	17	46	2	8	15	19	3	1438	1,355,108
30-	10	35	1	8	34	18	111	3	14	15	38	4	2038	1,339,658
35-	6	39	0	19	66	22	180	9	26	20	81	5	2590	1,153,155
40-	3	51	0	41	94	19	239	4	15	28	84	3	2580	846,773
45-	4	75	0	78	170	49	314	11	39	35	98	2	3352	855,146
50-	3	85	0	147	265	65	383	17	66	45	136	1	4671	688,043
55-	7	73	1	199	394	75	399	17	65	55	154	1	5681	563,297
60-	16	91	0	285	459	102	411	16	116	88	175	7	7464	484,754
65-	21	95	2	259	470	100	410	23	111	119	156	7	9432	382,483
70-	32	79	0	280	512	92	338	17	121	141	144	15	11,439	281,429
75-	39	39	0	180	352	82	213	17	110	128	101	6	10,555	179,351
80-	39	29	0	109	222	57	137	5	79	103	63	4	8864	109,024
> 85	33	15	0	65	127	54	70	0	62	57	33	7	7700	66,137

Diseases classified by ICD-10 code: A: diarrhea (A00, A01, A03, A04, A06-A09); B: viral hepatitis B (B16–B19 excluding B17.1, B18.2); C: viral hepatitis C (B17.1, B18.2); D: esophageal cancer (C15); E: stomach cancer (C16); F: colon/rectum cancer (C18–C21); G: liver cancer (C22); H: pancreatic cancer (C25); I: diabetes (E10–E14); J: ulcer (K25–K27); K: liver cirrhosis (K70, K74); L: appendicitis (K35–K37)

**Table 3:** Age-stratified number of deaths from major gastrointestinal diseases in 2011 in developed cities in China [14]

Age	Gastrointestinal diseases												All cause	Survival
	A	B	C	D	E	F	G	H	I	J	K	L		
0-	0	0	0	0	0	0	1	0	0	1	0	0	453	104,883
1-	1	0	0	0	0	0	0	0	0	0	0	0	123	404,828
5-	0	0	0	0	0	0	0	0	0	0	0	0	88	493,202
10-	0	0	0	0	0	0	0	0	1	0	0	0	85	516,918
15-	0	0	0	0	0	1	0	0	0	0	1	0	184	925,854
20-	0	0	0	0	4	0	2	1	2	1	1	0	363	1,024,382
25-	0	2	0	0	4	4	7	1	3	0	2	0	358	1,245,437
30-	0	3	0	0	12	9	26	3	13	0	5	0	463	1,049,958
35-	0	5	0	2	15	8	58	7	15	2	15	0	793	1,298,489
40-	0	14	0	10	44	31	119	12	15	3	35	0	1374	1,350,515
45-	0	20	1	46	107	63	212	35	43	5	72	0	2500	1,357,587
50-	0	31	1	67	154	92	288	54	65	7	81	0	3251	1,422,892
55-	0	32	2	125	242	157	359	97	101	14	82	0	4656	1,235,044
60-	1	27	2	114	252	170	353	92	147	12	64	1	4811	747,831
65-	0	12	2	138	270	145	270	107	167	11	57	0	5309	424,295
70-	1	21	5	137	339	239	285	133	284	25	50	1	8120	378,526
75-	1	31	3	162	469	335	354	151	433	24	63	2	11,978	279,355
80-	0	11	1	115	314	263	273	102	353	40	50	3	12,527	151,104
> 85	3	13	0	91	198	191	167	76	283	43	32	2	14,624	50,773

Diseases classified by ICD-10 code: A: diarrhea (A00, A01, A03, A04, A06-A09); B: viral hepatitis B (B16-B19 excluding B17.1, B18.2); C: viral hepatitis C (B17.1, B18.2); D: esophageal cancer (C15); E: stomach cancer (C16); F: colon/rectum cancer (C18–C21); G: liver cancer (C22); H: pancreatic cancer (C25); I: diabetes (E10-E14); J: ulcer (K25–K27); K: liver cirrhosis (K70, K74); L: appendicitis (K35–K37)

### Assessment of the role of urbanization and age in death caused by gastrointestinal disease

From the 2004 and 2011 data, we collected the age-stratified number of deaths from different gastrointestinal diseases (death group) and the age-stratified number of survivors (survival group) in the monitored urban and rural populations (Table 2 & Table 3). A generalized linear model (Poisson loglinear method) was used to assess the mortality risk from aging and urbanization with covariates of categorical age (age < 60 and age ≧ 60 for death group and survival group) and region (urban and rural); the cutoff of age was 65 years for appendicitis, because of the zeros for people aged under 60 in urbanised areas. The B coefficients for both age and region and interaction between region and age were obtained from the model: here the exp.(B) is interpreted as the risk ratio (RR). B = 0 indicated that given cause had no effect on the death; the greater (positive) the B, the greater the impact of urbanized factors or aging factor or effect of aging factor with urbanization on the gastrointestinal diseases.

Data were considered statistically significant when the probability of a type I error was 0.05 or less. Calculations were performed using the Windows version of SPSS 17.0 (SPSS Inc., Chicago, IL, US).

### Characteristics of the effects of aging and urbanized factors on gastrointestinal disease

The B value of interaction between region (cities and rural areas) and age was considered as indicator to assess role of age in mortality from gastrointestinal disease with urbanization, where a larger B value of interaction between region and age indicated a stronger effect of aging or a weaker effect of other risk factors in death with urbanization.

The quantitative values (B) of the impact of age on disease with urbanization were plotted against those for urbanized factors to form a scatter diagram. We used the locations of the various gastrointestinal diseases in this coordinate system to sort them according to the effects of aging and urbanization.

The group in the upper right quadrant represents old disease associated with the old population and developed urbanized disease; the group in the upper left quadrant shows disease associated with the other factor and developed urbanized disease; the group in the lower left quadrant, was termed other factor and undeveloped rural disease; the group in the lower right quadrant represents disease associated with the old population and undeveloped rural disease.

## Results

Generalized linear model was used to assess mortality risk from major gastrointestinal diseases with urbanization and aging as shown in Table 4. The contribution of urbanization to different gastrointestinal diseases varied; the B for the 12 gastrointestinal diseases ranged from − 5.760 to 1.216. A higher B indicated greater risk of disease with urbanization (negative value implied a protective effect). The Bs were, in ascending order, for gastrointestinal diseases of the diarrhea, appendicitis, ulcer, hepatitis B, liver cirrhosis, esophagus cancer, stomach cancer, liver cancer, diabetes, colon/rectum cancer, hepatitis C and pancreas cancer.

**Table 4:** Mortality risk from major gastrointestinal diseases with urbanization and aging

Diseases	Effect of region			Effect of age			Age*Region		
	B	Confidence interval(95%)	*p*	B	Confidence interval(95%)	*p*	B	Confidence interval(95%)	*p*
Diarrhea	−5.760	−7.723~ − 3.797	< 0.001	1.511	1.329~ 1.693	< 0.001	2.065	− 0.059~ 4.190	0.057
Hepatitis B	−1.274	−1.487~ − 1.060	< 0.001	1.983	1.839~ 2.129	< 0.001	− 0.127	− 0.427~ 0.174	0.409
Hepatitis C	0.720	−0.977~ 2.418	0.405	2.105	0.145~ 4.065	0.035	0.858	−1.399~ 3.116	0.456
Esophagus Ca.	−0.662	− 0.841~ − 0.510	< 0.001	2.966	2.861~ 3.071	< 0.001	− 0.073	− 0.251~ 0.104	0.417
Stomach Ca.	−0.559	−0.660~ − 0.458	< 0.001	2.822	2.748~ 2.895	< 0.001	0.115	−0.004~ 0.234	0.058
Colon/rectum Ca.	0.296	0.140~ 0.452	< 0.001	2.662	2.515~ 2.809	< 0.001	0.425	0.238~ 0.613	< 0.001
Liver Ca.	−0.438	− 0.514~ − 0.362	< 0.001	2.027	1.958~ 2.095	< 0.001	0.220	0.117~ 0.323	< 0.001
Pancreas Ca.	1.216	0.936~ 1.495	< 0.001	2.303	1.972~ 2.633	< 0.001	0.629	0.263~ 0.994	0.001
Diabetes	0.095	−0.080~ 0.271	0.287	3.015	2.866~ 3.165	< 0.001	0.635	0.436~ 0.834	< 0.001
Ulcer	−1.870	−2.236~ − 1.504	< 0.001	3.166	3.013~ 3.320	< 0.001	0.165	−0.241~ 0.571	0.425
Liver cirrhosis	−0.733	−0.872~ − 0.595	< 0.001	2.171	2.062~ 2.280	< 0.001	− 0.314	−0.507~ − 0.122	0.001
Appendicitis	−3.490	−5.480~ − 1.501	0.001	2.689	2.226~ 3.153	< 0.001	1.680	−0.450~ 3.810	0.122

Age*Region represents interaction between age and region; Ca. represents cancer

The quantitative values of the impact of age with urbanization on diseases also varied. The value of B (interaction between region and age) for the 12 gastrointestinal diseases ranged from − 0.314 to 2.065 as shown in Table 4. The larger the B value, the stronger the effect of aging with urbanization (negative value implied a protective effect from aging). The B values were, in ascending order, for liver cirrhosis, hepatitis B, esophagus cancer, stomach cancer, ulcer, liver cancer, colon/rectum cancer, pancreas cancer, diabetes, hepatitis C, appendicitis and diarrhea.

**Fig. 1:**
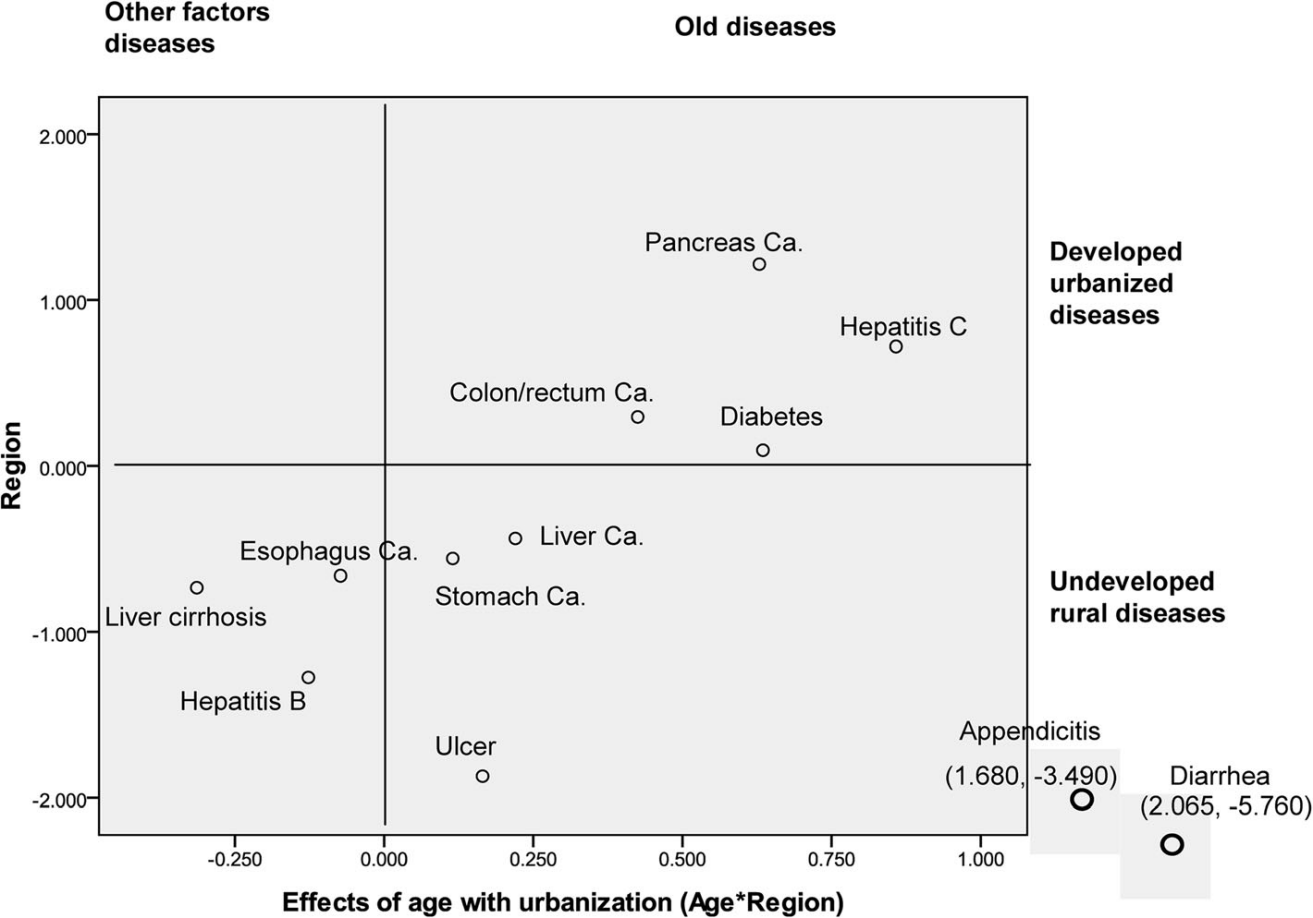
Gastrointestinal disease groups according to sensitivity to urbanization and aging

The effects of age and urbanization on gastrointestinal disease are summarized and quantified in Fig. 1. According to their location on the scatter diagram, the 12 gastrointestinal diseases could be divided into three groups. In the upper right quadrant is what we termed old and developed urbanized disease, which had 4 diseases. The second group, closer to the lower left quadrant, was termed other factor and undeveloped rural disease, and had 3 diseases. The third group close to the lower right quadrant, was termed old and undeveloped rural disease and had 5 diseases.

## Discussion

In our study, the life expectancy at birth was 79.43 years in urban areas and 73.90 years in rural areas, implying that data from urban areas and rural areas can be used for determining sensitivity to urbanization. Urbanization is associated with elongated life expectancy and thus higher rate of diseases that occur in elderly group; that aging and urbanization were included in one model as covariates could be eliminated the influence of elongated life expectancy for analyzing effects of urbanization on diseases.

Nutritional deficiencies could be referred to as an undeveloped rural disease. In present study, the mortality due to nutritional deficiencies was higher in rural areas than that in urban areas, implying that data sets were reliable. Although the diagnostic technique could be improved with urbanization, the rates of objective diagnosis (as evidenced by laboratory, pathologic, imaging and/or surgical intervention findings) were almost same in two regions, suggesting that bias from the improved diagnostic technique could be limited.

The B value was used to quantify the contribution of changes in urbanization to mortality from gastrointestinal disease revealed that these contributions varied. A higher B indicated greater risk of disease with urbanization (negative value implied a protective effect). We defined gastrointestinal diseases with a value of B (region) greater than zero as the “developed urbanized disease”, and from our analysis, four gastrointestinal diseases (diabetes, colon/rectum cancer, hepatitis C and pancreas cancer) were developed urbanized disease. Urbanization may be a protective factor for other gastrointestinal diseases.

It is well known that the incidence of diabetes mellitus increases greatly with the development of a society [16–18]. Therefore, diabetes mellitus could be referred to as a developed urbanized disease. In the present study, diabetes mellitus was included in a group of developed urbanized disease, implying that statistical method was appropriate in our study.

As aging is a key observed variable for non-communicable diseases [19] and an uncontrollable factor in mortality, it is important to evaluate the effects of aging on gastrointestinal disease with urbanization. The B value of interaction between age and region (cities and rural areas) could be considered as indicator to assess role of age in mortality from gastrointestinal disease with urbanization, where a larger B value implied a stronger effect of aging with urbanization; the negative B value implied a stronger effect of other urbanized factors except aging in death with urbanization. We found that the value of B (interaction between region and age) for the 12 gastrointestinal diseases also varied.

We defined gastrointestinal diseases with a value of B (interaction between region and age) greater than zero as the “old diseases”, and from our analysis, nine gastrointestinal diseases (stomach cancer, ulcer, liver cancer, colon/rectum cancer, pancreas cancer, diabetes, hepatitis C, appendicitis and diarrhea) were old diseases; for these diseases, aging could become a major cause of death with urbanization. This indicates that common protective interventions for health or aging process have an important role.

The scatter diagram represented the effects of aging and urbanization on gastrointestinal disease together as the dividing value. We divided the 12 gastrointestinal diseases into three groups according to their location on the diagram; each group represented gastrointestinal diseases with different attributes.

The group in the lower left quadrant, was termed other factor and undeveloped rural disease and includes esophageal cancer, liver cirrhosis and hepatitis B. Urbanization may be a protective factor for this group of diseases. We also clearly saw a tendency toward other factor (non-aging factors) with the development of society and the increase in the life expectancy, suggesting a independent of aging and a characteristic of programmed onset (occurrence of disease in a certain life stage) [20]. Accordingly, an emphasis on increasing basal health status may contribute to disease preventative strategies in this group.

The group in the lower right quadrant represents disease associated with the old population and undeveloped rural areas and includes stomach cancer, liver cancer, gastrointestinal ulcers, diarrhea and appendicitis. Urbanization may also be a protective factor for this group of diseases. Undeveloped rural risk factors for this disease group appear to be eliminated with urbanization. Therefore, aging will be a major causes of death with urbanization for this group of diseases. Accordingly, an emphasis on public health may contribute to disease preventative strategies in this group.

The group in the upper right quadrant shows disease associated with the relative-old onset and developed urbanized disease and included pancreas cancer, colon/rectum cancer, hepatitis C, and diabetes. That disease occurs relative-old implies the increase of age at disease onset is so many as the increase of life expectancy. The aging and increase of life expectancy may be major causes for mortality, suggesting this group of diseases should be prevented by delaying aging, although it may be difficult.

The group in the upper left quadrant shows disease associated with the other factor and developed urbanized disease; unexpectedly, there is not major gastrointestinal diseases in this group. This group disease implies the increase of age at death is not so many as the increase of life expectancy. The other risk factors (non-aging factors) from urbanization, such as sedentary lifestyle and higher-kilojoule food intake [21–23], may be major causes for mortality, suggesting this group of diseases could be prevented by avoiding these urbanization associated risk factors. Further study is needed to explore whether these risk factors from urbanization have important role in occurrence of some gastrointestinal diseases or not.

## Conclusion

Most gastrointestinal diseases were sensitive or protective to change of socioenvironmental factors; death from gastrointestinal disease would increase with the urbanization for some of the types and would decrease with the urbanization for the other types. Since the aging factors leading to disease are relatively uncontrollable, the study and control of external risk factors could lead to the conversion of most gastrointestinal disease. Common protective interventions for public health and quality of life, which include quality of social life and nutritional status, have the potential to eliminate the undeveloped rural diseases. Understanding the categorization of gastrointestinal disease according to urbanization and age will aid in its primary prevention.

## References

[1] Zeng Z, Zhu Z, Yang Y, Ruan W, Peng X, Su Y, Peng L, Chen J, Yin Q, Zhao C, Zhou H, Yuan S, Hao Y, Qian J, Ng SC, Chen M, Hu P (2013). Incidence and clinical characteristics of inflammatory bowel disease in a developed region of Guangdong Province, China: a prospective population-based study. J Gastroenterol Hepatol.

[2] Amorim CA, Moreira JP, Rial L, Carneiro AJ, HS FÃ§a, Elia C, Luiz RR, de Souza HS (2014). Ecological study of gastric cancer in Brazil: geographic and time trend analysis. World J Gastroenterol.

[3] Wong SH, Ng SC (2013). What can we learn from inflammatory bowel disease in developing countries?. Curr Gastroenterol Rep.

[4] Liang Y, Li S (2014). Landless female peasants living in resettlement residential areas in China have poorer quality of life than males: results from a household study in the Yangtze River Delta region. Health Qual Life Outcomes.

[5] Chen J (2013). Chronic conditions and receipt of treatment among urbanized rural residents in China. Biomed Res Int.

[6] Wu N, Tang X, Wu Y, Qin X, He L, Wang J, Li N, Li J, Zhang Z, Dou H, Liu J, Yu L, Xu H, Zhang J, Hu Y, Iso H (2014). Cohort profile: the Fangshan cohort study of cardiovascular epidemiology in Beijing, China. J Epidemiol.

[7] Ma L, Mai J, Jing J, Liu Z, Zhu Y, Jin Y, Chen Y (2014). Empirical change in the prevalence of overweight and obesity in adolescents from 2007 to 2011 in Guangzhou, China. Eur J Pediatr.

[8] Zhang Y, Mo J, Weschler CJ (2013). Reducing health risks from indoor exposures in rapidly developing urban China. Environ Health Perspect.

[9] Chan F, Adamo S, Coxson P, Goldman L, Gu D, Zhao D, Chen CS, He J, Mara V, Moran A (2012). Projected impact of urbanization on cardiovascular disease in China. Int J Public Health.

[10] Hui L (2015). Chronic diseases and societal development, based on the death-risk index. Epidemiology.

[11] Gong P, Liang S, Carlton EJ, Jiang Q, Wu J, Wang L, Remais JV (2012). Urbanisation and health in China. Lancet.

[12] Idrovo AJ (2011). Physical environment and life expectancy at birth in Mexico: an eco-epidemiological study. Cad Saude Publica.

[13] Chinese Center for Disease Control and Prevention. National Disease Mortality Surveillance System, 2004. Military Medical Science Press, 2009:102–107.

[14] Chinese Center for Disease Control and Prevention. National Disease Mortality Surveillance System, 2011. People’s Medical Publishing House, 2013:171–405.

[15] World Health Organization (1992). International statistical classification of diseases and related health problems, tenth revision.

[16] Xin G, Yang G, Hui L (2014). Study to assess whether waist circumference and changes in serum glucose and lipid profile are independent variables for the CETP gene. Diabetes Res Clin Pract.

[17] Sherwin R, Jastreboff AM (2012). Year in diabetes 2012: the diabetes tsunami. J Clin Endocrinol Metab.

[18] Xin G, Shong L, Hui L (2014). Effect of genetic and non-genetic factors, including aging, on waist circumference and BMI, and inter-indicator differences in risk assessment. Exp Gerontol.

[19] Hui L (2017). Assessment of the role of ageing and non-ageing factors in death from non-communicable diseases based on a cumulative frequency model. Sci Rep.

[20] Hui L (2015). Aging and chronic disease as independent causative factors for death and a programmed onset for chronic disease. Arch Gerontol Geriatr.

[21] Doherty ML, Owusu-Dabo E, Kantanka OS, Brawer RO, Plumb JD (2014). Type 2 diabetes in a rapidly urbanizing region of Ghana, West Africa: a qualitative study of dietary preferences, knowledge and practices. BMC Public Health.

[22] Ng SW, Howard AG, Wang HJ, Su C, Zhang B (2014). The physical activity transition among adults in China: 1991-2011. Obes Rev.

[23] Du SF, Wang HJ, Zhang B, Zhai FY, Popkin BM (2014). China in the period of transition from scarcity and extensive undernutrition to emerging nutrition-related non-communicable diseases, 1949-1992. Obes Rev.

